# Cytoadherence in paediatric malaria: ABO blood group, CD36, and ICAM1 expression and severe *Plasmodium falciparum* infection

**DOI:** 10.1111/bjh.12014

**Published:** 2012-08-22

**Authors:** Christine M Cserti-Gazdewich, Aggrey Dhabangi, Charles Musoke, Isaac Ssewanyana, Henry Ddungu, Deborah Nakiboneka-Ssenabulya, Nicolette Nabukeera-Barungi, Arthur Mpimbaza, Walter H Dzik

**Affiliations:** 1 University Health Network/University of TorontoToronto, ON, Canada; 2 Mulago Hospital/Makerere University College of Health SciencesKampala, Uganda; 3 Central Public Health Laboratories (CPHL)Kampala, Uganda; 4 Uganda Cancer Institute & the African Palliative Care Association (APCA)Kampala, Uganda; 5 London School of Hygiene and Tropical MedicineLondon, UK; 6 Massachusetts General Hospital/Harvard UniversityBoston, MA, USA

**Keywords:** Malaria, ABO, Anaemia, Platelet, CD36, ICAM1

## Abstract

As a leading cause of childhood mortality worldwide, selection pressure by *Plasmodium falciparum* continues to shape the human genome. Severe disturbances within the microcirculation result from the adhesion of infected erythrocytes to host receptors on monocytes, platelets, and endothelium. In this prospective study, we compared expression of all major host cytoadhesion receptors among Ugandan children presenting with uncomplicated malaria (*n* = 1078) versus children with severe malaria (*n* = 855), including cerebral malaria (*n* = 174), severe anaemia (*n* = 522), and lactic acidosis (*n* = 154). We report a significant survival advantage attributed to blood group O and increased monocyte expression of CD36 and ICAM1 (CD54). The high case fatality rate syndromes of cerebral malaria and lactic acidosis were associated with high platelet CD36 expression and thrombocytopenia, and severe malaria anaemia was characterized by low ICAM1 expression. In a logistic regression model of disease severity, odds ratios for the mitigating effects of blood group O, CD36, and ICAM1 phenotypes were greater than that of sickle haemoglobin. Host genetic adaptations to *Plasmodium falciparum* suggest new potential malaria treatment strategies.

Although the human genome has been remarkably altered by selective pressure from malaria (Weatherall, 2008), infection with *Plasmodium falciparum* continues to kill large numbers of children born within malaria endemic regions characterized by limited health resources (World Health Organization [WHO], 2011). Prior to developing adaptive immunity and gaining access to therapy, malaria-infected children depend upon host attributes that optimize parasite clearance or mitigate lethal pathophysiology (Stevenson & Riley, 2004). The best appreciated adaptations to *P. falciparum*, such as sickle haemoglobin and thalassaemia, appear to be concerned not with invasion resistance, but rather with earlier clearance of infected erythrocytes (iRBC) within the spleen as a result of modified red cell fitness (Modiano, 1998). Furthermore, the most lethal malaria syndromes appear to associate with enhanced iRBC adhesion (Miller *et al*, 2002; Rowe *et al*, 2009a), wherein parasite traffic to the spleen is reduced by systemic (“extrasplenic”) sequestration of iRBC. Although favourable to survival of the parasite, adhesion and sequestration of iRBC result in microvascular obstruction and tissue hypoperfusion in the host (van der Heyde *et al*, 2006). Whether human hosts show any evidence of reduced or differential expression of adhesion receptors (Cserti-Gazdewich *et al*, 2011) as a counter-adaptation to iRBC cytoadhesion has not been fully investigated and is the focus of this study.

The most appealing candidates for possible co-evolution are host receptors of adhesion with *P. falciparum* Erythrocyte Membrane Protein (PfEMP-1) (Cooke *et al*, 2004). In laboratory studies, erythrocytes expressing blood group A1, the primordial human ABO type (Calafell *et al*, 2008), exhibit strong rosette formation through binding of the lectin-like domain of PfEMP-1 (Carlson & Wahlgren, 1992; Vigan-Womas *et al*, 2012), while the evolutionarily more recent group O erythrocytes rosette least (Rowe *et al*, 2007). PfEMP-1 contains alternative domains that bind the host receptors ICAM1 (CD54) and CD36. It has been proposed that their expression on monocytes may benefit the host by mediating immune clearance of iRBCs (McGilvray *et al*, 2000; Baratin *et al*, 2007). However, ICAM1 and CD36 are also expressed on endothelial cells, promote adhesion of iRBC to the vasculature (Yipp *et al*, 2000), and therefore may be detrimental to the host. The biology of sequestration via CD36 is further complicated by its expression on platelets (as platelet glycoprotein IV). Platelets expressing CD36 may mediate microvascular occlusion either by facilitating clumping of iRBC (Pain *et al*, 2001) or by bridging adhesion of iRBC to endothelial cells (Bridges *et al*, 2010).

Consistent with human-parasite co-evolution, prior studies have observed a higher ratio of blood group O-to-A (Cserti & Dzik, 2007) and a higher prevalence of ICAM1 (Fernandez-Reyes *et al*, 1997) and CD36 (Gelhaus *et al*, 2001; Fry *et al*, 2009) mutations among individuals who reside in areas endemic for *P. falciparum*. However, prior clinical studies have either examined cytoadhesion receptors individually, not accounted for the effect of other clinical risk factors, or have had small sample sizes. As a result, the relative contribution of the major host cytoadhesion receptors to malaria pathogenesis has remained incompletely characterized (Rowe *et al*, 2009b; Ochola *et al*, 2011).

To test the hypothesis that host blood cell phenotypes are associated with malaria severity, we conducted the Cytoadherence in Paediatric Malaria (CPM) Study in a cohort of two thousand children in Uganda. We compared expression of the major host cytoadhesion receptors across a spectrum of malaria severity, quantified their relative effect size compared with other recognized factors that influence severity (such as sickle trait), and examined their distributions in three severe malaria syndromes.

## Methods

### Study population

This prospective case-control study enrolled children, age 6 months to 12 years, presenting with either initially severe or uncomplicated *P falciparum* malaria to Mulago Hospital's Acute Care Unit in meso-endemic Kampala, Uganda between October 15, 2007 and October 30, 2009. Children with severe malnutrition were ineligible for enrolment. The malaria diagnosis was established first with clinical symptoms and a positive screening thick smear. Two blinded reviewers of thin and thick smears at a reference parasitology laboratory independently confirmed the diagnosis and parasite density using leucocyte counts (Figs 2 in supporting information).

**Fig. 1:**
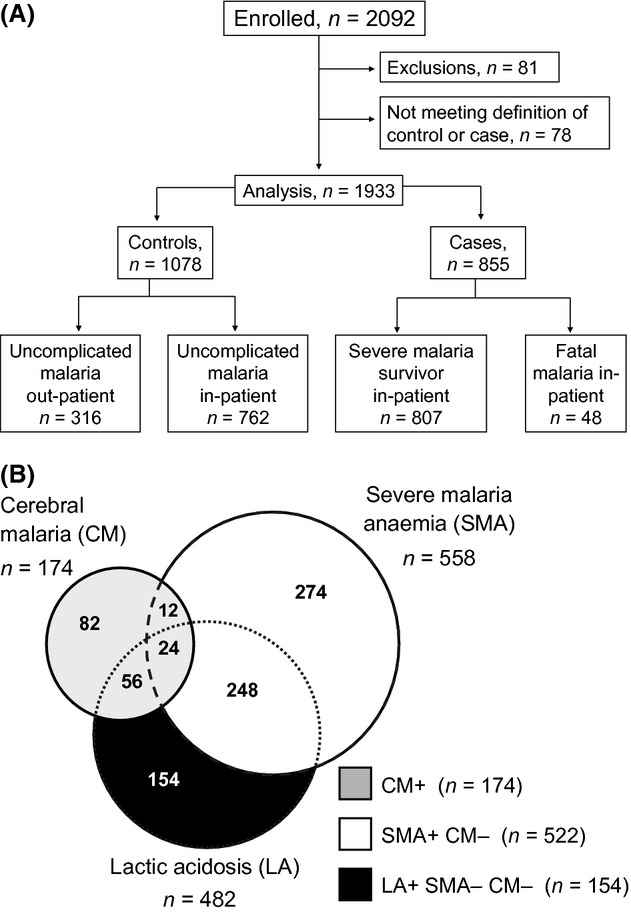
Study design and patient categories. (A) Pre-specified categories of patients enrolled. The analysis of severity of disease compares controls (uncomplicated malaria, UM, *n* = 1078) with cases (severe malaria, SM, *n* = 855). (B) Distribution of the three principal severe malaria syndromes among the cases. For analysis, cases are classified into three non-overlapping categories: all cerebral malaria (CM) (grey); severe malaria anaemia (SMA) without CM (white); and isolated lactic acidosis (LA) not attributable to SMA or CM (black).

**Fig. 2:**
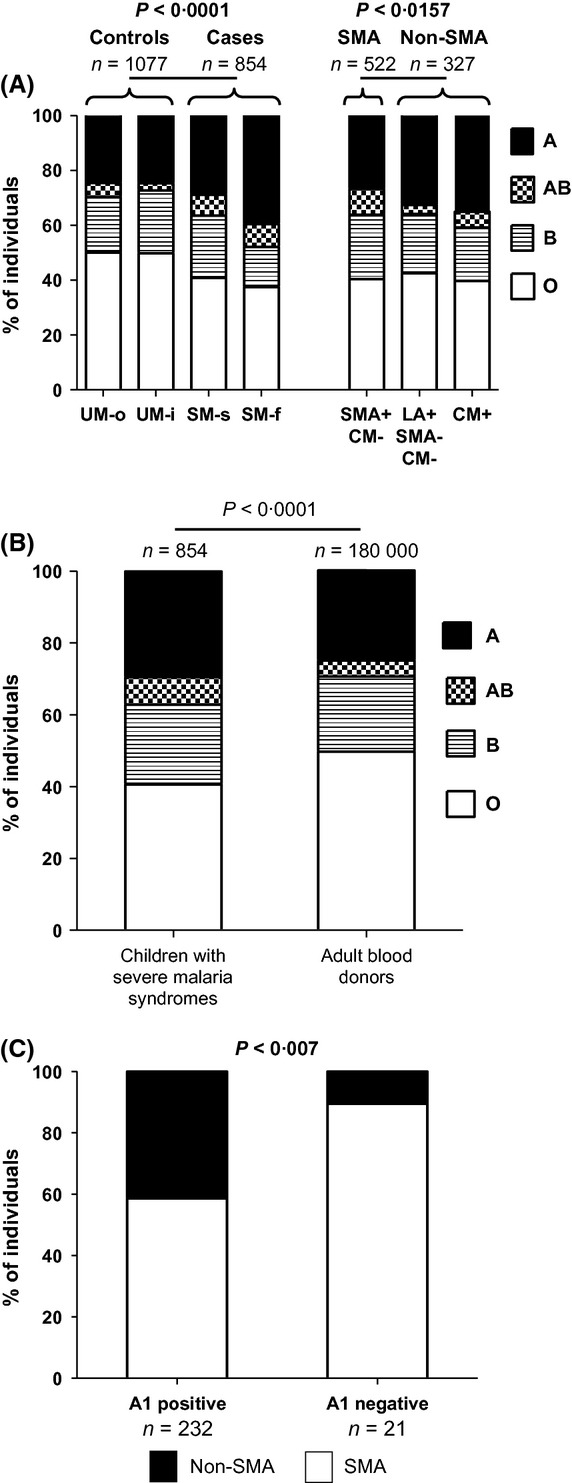
ABO blood groups in patients with malaria. (A) ABO groups according to the spectrum of disease severity. Controls have a higher prevalence of group O, and cases have a higher prevalence of non-group O. (B) ABO distributions among the cases (severe or fatal malaria) are significantly different from that observed in healthy adult Ugandan blood donors. (C) Among group A or AB cases, A1 positive cases are more likely to have CM or LA than those negative for A1. UM-o, uncomplicated malaria treated as an outpatient; UM-i, uncomplicated malaria treated as an inpatient. SM-s, severe malaria, survivor; SM-f, severe malaria, fatal; SMA, severe malaria anaemia; LA, lactic acidosis; CM, cerebral malaria.

### Oversight

Approval for the study was granted by Makerere University Faculty of Medicine Research Ethics Committee, the Toronto Academic Health Science Network Research Ethics Board, and the Uganda National Council for Science and Technology. The study was registered as NCT 00707200 at http://www.clinicaltrials.gov.

### Consent form

The consent form was available in both English and Luganda and was approved by research ethicists in Toronto, Canada and Kampala, Uganda, in accordance with Good Clinical Practice Guidelines. The research officers, fluent in both languages, were trained in the administration and documentation of informed consent from the parents or legal guardians of paediatric subjects. Parents/guardians retained a copy of consent forms with supplementary study summary pamphlets and investigator contact information.

### Data collection

Each enrolled patient was evaluated and treated by physicians experienced in malaria care, using all available clinical resources to assess the presence of other conditions. Data were recorded on a hard-copy Case Report Form (CRF), available at http://www.cd36malaria.org. After each subject's discharge, data were transferred to a digital CRF. Regular fortnightly conference calls (*n* = 55) reviewed logistics, tabulated accrual statistics, and monitored study quality control. The accuracy of data transfer to the digital CRF was audited on each 25th CRF quarterly. The digital CRF (prepared in FileMaker Pro 9·0 v 1, Santa Clara, CA, USA) mirrored the paper CRF with multiple features to prevent transcription errors. Paper and digital CRFs were kept in a secure, locked environment during enrollment and transferred at the conclusion of enrollment to study headquarters for use in resolution of any discrepancies in the database.

The electronic study database, prepared from exports of the digital CRF, underwent extensive testing for data integrity, consistency, and accuracy. Scrutiny included range value testing, missing data testing, logical tests on data consistency across fields, and multiple comparisons with the original paper CRF record. Strict version control was used. All data analyses were done on a single version of a finalized corrections dataset.

### Clinical procedures and categorization

Patients were observed until discharge or death by two study physicians. Prior to analysis, each patient was categorized at final disposition as a case or control, and if failing to meet pre-specified definitions, was excluded. Controls were those with uncomplicated malaria (UM), defined as the absence of any impairment of consciousness or hypoxia, with peripheral blood lactate <5 mmol/l and haemoglobin (Hb) >70 g/l without transfusion. Cases were those with severe malaria (SM) including severe malaria anaemia (SMA), defined as Hb < 50 g/l (or <60 g/l after transfusion); lactic acidosis (LA), defined as blood lactate >5 mmol/l; hypoxia, defined as oxygen saturation <90% while breathing ambient air; or cerebral malaria (CM), as defined below.

### Definition of cerebral malaria (CM)

WHO guidelines provide general criteria to characterize cerebral malaria, i.e., Blantyre Coma Scale (BCS) ≤2 with depressed levels or loss of consciousness (LOC) persisting >1 h after any possible prior convulsions (Idro *et al*, 2005). Due to recent evidence for the non-specificity of WHO criteria for CM when compared with autopsy confirmation or expert retinoscopy (Taylor *et al*, 2004), and because the neurological literature on LOC has established that prolonged post-ictal states and/or anticonvulsants may themselves affect level of consciousness, we opted for a stricter screening definition of CM that accepted coma under the following conditions:

1. Coma was present for more than 6 h, particularly if following convulsions, and
2. Coma was not attributable to high fevers, hypoglycaemia, meningitis, non-malaria-related pre-existing neurological abnormalities, or drugs, such as anticonvulsants or other agents with sedative/hypnotic effects (WHO, 1986).

To increase the specificity of our categorization of CM cases, we used objective clinical criteria that included both WHO features of severe disease, and a case report form-derived scoring system. A case was designated as probable to definite CM (*n* = 174) if the patient had coma plus >3 of the following 10 WHO malaria severity criteria: >2 seizures in 24 h, respiratory distress, jaundice, haemoglobinuria, spontaneous bleeding, hypoglycaemia (glucose < 2·2 mmol/l), lactic acidosis (lactate >5 mmol/l), normocytic severe anaemia, hyperparasitaemia >5%, or new acute renal failure; or if the patient had a cumulative score of ≥3 points (*n* = 169) using the following scale:

1. Coma:
  1 point: survivor with timing of coma to seizures not specified.
  2 points: fatal case with timing of coma to seizures not specified.
  3 points: coma present >6 h after last seizure.
2. Blantyre Score <3:
  1 point: survivor with timing of Blantyre score to seizures not specified.
  2 points: fatal case with timing of Blantyre score to seizures not specified.
  3 points: score <3 and performed >6 h after last seizure.
3. Seizures witnessed in hospital:
  1 point: survivor with ≤3 observed seizures (or number not recorded) in 24 h.
  2 points: fatality with ≤3 observed seizures (or number not reported) in 24 h.
  6 points: >3 observed seizures in 24 h.
4. Blood sugar:
  0 points: blood sugar not tested at time of coma or Blantyre assessment.
  0 points: blood sugar ≥3 mmol/l at time of coma or Blantyre score assessment.
  −1 point: blood sugar <3 mmol/l at time of coma or Blantyre score assessment.
5. Lumbar puncture (performed in *n* = 56):
  0 points: not performed.
  1 point: performed and did not show signs of meningitis.
6. Retinoscopy (performed in *n* = 12):
  0 points: if negative or not performed.
  2 points: if positive.

### Laboratory procedures

Patients were excluded if human immunodeficiency virus (HIV) positive. 740 patients were tested for HIV in Kampala using a screening assay (Clearview® STAT-PAK®, Inverness Medical, Louisville CO, USA). Negative screening tests were re-tested with a second assay (Uni-Gold™ Recombigen® HIV, Trinity Biotech PLC, Wicklow, Ireland), and positive screening tests confirmed with a second enzyme-linked assay (Murex HIV Ag/Ab Combination; Abbott Diagnostics Division, Dartford, UK; or Vironistika® HIV Uni-Form II Ag/Ab; bioMérieux, Lyon, France). Archived plasma aliquots from the remaining 1331 who were not tested in Kampala (or not already excluded) were tested with a high-sensitivity commercial assay for antigen and antibody at Mount Sinai Hospital's Microbiology Laboratory in Toronto, Canada (Architect System HIV Ag/Ab Combo; Abbott Diagnostics Division, Weisbaden, Germany). 45 HIV+ subjects were excluded from analysis. Of the 733 subjects identified as HIV-negative by laboratory testing in Kampala, 0·5% of samples were randomly re-assessed in Toronto to validate the negative result.

ABO blood groups were determined locally on peripheral blood collected in EDTA- anticoagulated tubes. ABO Blood Group Reagents (Anti-A BioClone®, Anti-B BioClone®, Anti-D for Slide and Modified Tube Tests, ORTHO® Anti-A1 Lectin; Ortho-Clinical Diagnostics, Inc, Raritan, NJ, USA) were applied to whole blood for direct haemagglutination typing according to manufacturer's directions.

Monocyte CD54, monocyte CD36, and platelet CD36 were determined in a blinded fashion by flow cytometry (Joint Clinical Research Centre, Kampala) according to previously published methods established for malaria patients (Cserti-Gazdewich *et al*, 2009, 2010; Dzik *et al*, 2010) measuring median fluorescence intensity (MFI) (Fig 3 in supporting information). Values for monocyte CD36 MFI were corrected for the effect of platelet count prior to analysis (Dzik *et al*, 2010). Complete blood counts were determined by Coulter AC*T8 (Coulter Corp, Miami, FL, USA). Peripheral blood lactate was measured with a point-of-care device (Lactate-Pro, Arkray Inc, Kyoto, Japan). Oxygen saturation was measured by finger oximetry (Nonin Corp, Plymouth, MN, USA). The presence of haemoglobin S (HbS) was detected by solubility (SickleDex®, Streck Laboratories, Omaha, NB, USA).

**Fig. 3:**
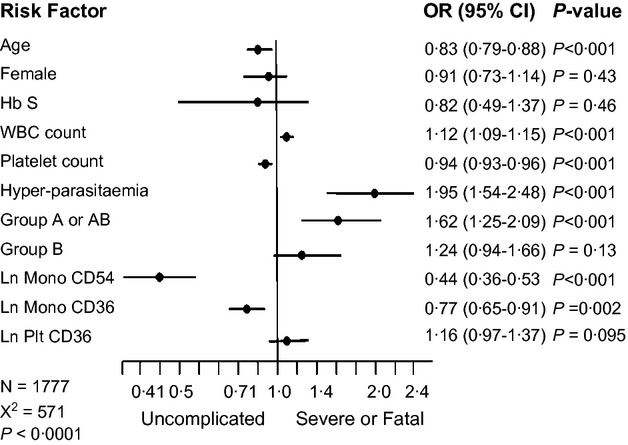
Factors associated with uncomplicated or severe malaria. Results of logistic regression model showing the odds ratios (OR, black circles) and 95% confidence intervals (95% CI, horizontal lines) for uncomplicated versus severe or fatal malaria according to input risk factors. Odds ratios for age are scaled per year and for white blood cell count (WBC) and platelet count (Plt) are scaled in increments of 10^9^/l and 10 × 10^9^/l, respectively. Mono, monocyte; Hb S, haemoglobin S.

### Statistical analysis

Continuous data are reported as a median with inter-quartile ranges, and compared using a Wilcoxon test with α = 0·05. Categorical data were compared using the chi-square test with α = 0·05. All comparisons were two tailed.

Logistic regression was used to measure factors associated with severe malaria. Input variables in the model were age, sex, presence of haemoglobin S, leucocyte count, platelet count, presence of hyperparasitaemia, ABO group, monocyte CD54, monocyte CD36 and platelet CD36 (Table I in supporting information). Because group A and AB individuals strongly express the A antigen, they were entered together in the model. Because CD36 and CD54 MFI approximate a log-normal distribution (Fig 4 in supporting information), log-transformed values were entered in the model. Odds ratios (OR) and significance were computed using commercial software (Stata® ver 11.1; Stata Corp, College Station, TX, USA).

**Fig. 4:**
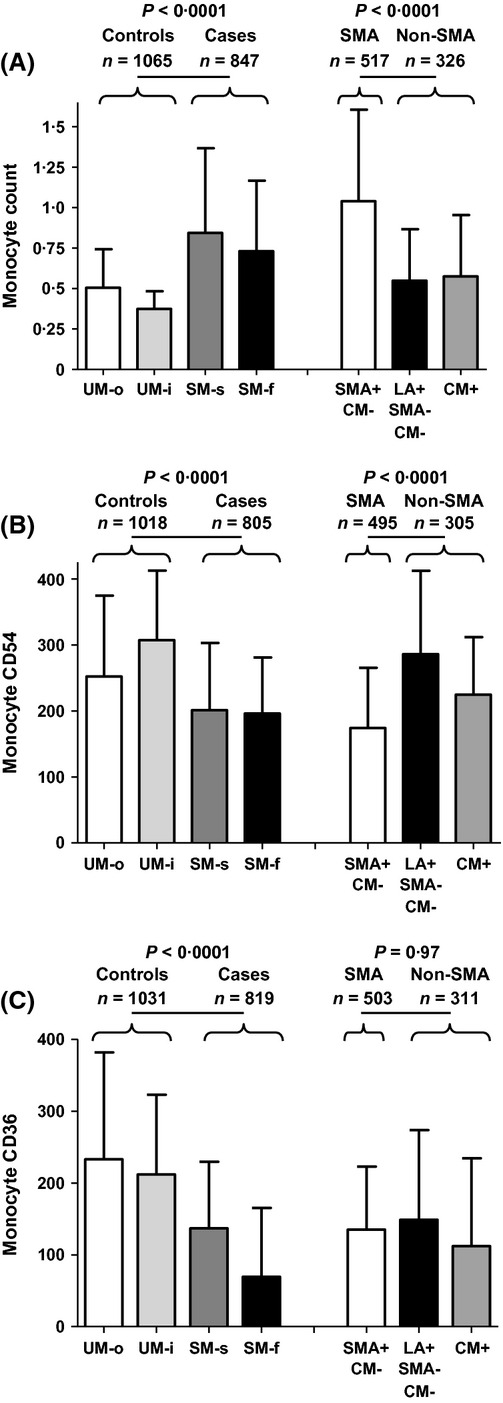
Monocyte CD54 and CD36 expression in patients with malaria. (A) The median and inter-quartile range of monocyte counts is shown. (B) The median and inter-quartile range of CD54 (ICAM1) expression on monocytes (measured as median fluorescence intensity using flow cytometry). Low monocyte CD54 expression is seen among cases with SMA. (C) The median and inter-quartile range of CD36 expression on monocytes (measured as median fluorescence intensity using flow cytometry). A progressive decline in monocyte CD36 expression is associated with categories of increasing disease severity. UM-o, uncomplicated malaria treated as an outpatient; UM-i, uncomplicated malaria treated as an inpatient; SM-s, severe malaria; survivor. SM-f, severe malaria, fatal; SMA, severe malaria anaemia; LA, lactic acidosis; CM, cerebral malaria.

The study was powered to detect a difference in the proportion of cases with blood group A or AB compared with the proportion of controls with group A or AB. Assuming a baseline prevalence of group A or AB ranging from 0·25 to 0·4, our study, with approximately 1000 cases and 1000 controls, could detect a 6% or greater difference in the proportion of group A or AB among cases versus controls with at least 80% power and a type I error probability of 0·05.

## Results

### Patients

A total of 2092 patients were enrolled, with 81 exclusions (45 HIV+, 35 falsely positive for *P falciparum*, 1 not of predefined age-range). Of the 2011, 78 were excluded from analysis because at final disposition, they did not meet pre-study definitions of uncomplicated malaria (UM) or severe malaria (SM). Controls included UM outpatients (UM-o, *n* = 316, 29·1%) and UM inpatients (UM-i, *n* = 762, 39·4%). Cases comprised SM survivors (SM-s *n* = 807, 41·7%) and fatalities (SM-f, *n* = 48, 5·6%) (Fig 1A). Median hospital length of stay was 2, 3 and 1 d among UM-i, SM-s and SM-f, respectively.

Illness in the great majority was attributable exclusively to malaria rather than to other infections. None of the 56 lumbar punctures in suspected cases of cerebral malaria showed meningitis. In four chest radiographs of suspected pneumonia, only one showed infiltrates. Parenteral antibiotics were empirically offered in one case of unconfirmed meningitis and 38 cases of unconfirmed pneumonia.

Table I compares baseline patient features between UM and SM. Children with UM and SM had similar sex ratios and prevalence of haemoglobin S (HbS). Cases were significantly younger, had a lower body mass index, had been unwell for longer before hospital presentation, and had a greater extent of palpable splenomegaly. Although median parasitaemia was similar in cases and controls, hyperparasitaemia (>5% of erythrocytes parasitized) was present twice as often among cases (48% vs. 25%). Median (interquartile range) parasitaemia in children positive for HbS was half that of children negative for HbS [44 200 (16 800–141 000) vs. 88 400 (27 400–216 000), *P* = 0·0031]. As shown in Table I, SM was also characterized by lower platelet counts, a higher prevalence of severe thrombocytopenia, and higher leucocyte and monocyte counts.

**Table I:** Clinical and laboratory findings in children with *Plasmodium falciparum* malaria

	Comparison by severity (*n* = 1933)					Comparison by severe syndrome type (*n* = 850)						
		
	Controls *n* = 1078 uncomplicated malaria (UM)		Cases *n* = 855 severe malaria (SM)		*P* value UM vs. SM	Severe malaria anaemia (SMA)* *n* = 522		Isolated lactic acidosis (LA)† *n* = 154		Cerebral malaria (CM)‡ *n* = 174		
							
		*n*		*n*			*n*		*n*		*n*	*P* value SMA vs. non-SMA ¶
Patient demographic factors: median, [IQR] or n (%)												
Age (years)	2·9 [1·6–5·1]	1078	1·8 [1·1–3·1]	855	<0·0001	1·6 [1·0–2·6]	522	2·4 [1·2–3·9]	154	2·51 [1·5–3·9]	174	<0·0001
Body mass index	15·4 [14·0–17·0]	797	14·8 [13·6–16·5]	655	<0·0001	14·8 [13·6–16·2]	414	15·2 [14·1–17·7]	115	14·7 [13·3–16·7]	122	0·086
Sex (female: male)	521:557 (48:52)	1078	402:453 (47:53)	855	0·60	242:280 (46:54)	522	69:85 (45:55)	154	90:84 (52:48)	174	0·60
Presence of HbS	57 (6%)	1045	43 (5%)	826	0·89	30 (6%)	505	7 (5%)	149	6 (4%)	167	0·33
Malaria presentation features: median, [IQR] or n (%)												
Days unwell prior to hospitalization	3 [2–4]	1078	3 [3–5]	855	<0·0001	4 [3–5]	522	3 [2–4]	154	3 [3–4]	174	<0·0001
Palpable spleen below costal margin (cm)	0	674	2 [0–4]	504	<0·0001	3 [0–4]	307	0·75 [0–3]	108	2 [0–3]	87	0·0002
Haemoglobin (g/l)	93 [82–104]	1078	45 [36–63]	855	n/a	38 [32–44]	522	72 [59–87]	154	69 [52–82]	174	n/a
SMA (%)	0	1078	558 (65%)	855	n/a	522 (100%)	522	0 (0%)	154	36 (21%)	174	n/a
MCV (fl)	84 [78–89]	1077	84 [78–90]	855	0·61	85 [79–93]	522	83 [78–88]	154	84 [79–89]	174	0·039
Parasitized RBCs/μl	83 000 [29 000–190 000]	1063	91 100 [21 600-263 200]	831	0·13	67 600 [14 800–191 800]	503	187 000 [61 900–462 600]	152	124 000 [25 600– 386 800]	171	<0·0001
% RBCs parasitized	2·2 [0·8–5·0]	1062	4·6 [1·2–12·9]	831	<0·0001	4·2 [1·0–11·4]	503	6·6 [2·4–14·3]	152	4·6 [1·1–16·2]	171	0·039
Hyper-parasitaemia (>5% iRBC), (%)	264 (25%)	1063	402 (48%)	831	<0·0001	232 (46%)	503	83 (54%)	152	85 (50%)	171	0·11
Platelet count (×10^9^/l)	136 [81–217]	1078	103 [60–170]	854	<0·0001	123 [79–181]	522	78 [42–125]	154	73 [43–130]	174	<0·0001
Severe (<50 × 10^9^/l) thrombocytopenia	104 (10%)	1078	166 (19%)	854	<0·0001	60 (11%)	522	48 (31%)	154	57 (33%)	174	<0·0001
Leucocyte count (×10^9^/l)	7·8 [5·9–10·3]	1072	11·1 [7·7–16·7]	853	<0·0001	12·6 [8·6–18·7]	520	9·1 [6·6–11·5]	154	9·4 [7·1–14·3]	174	<0·0001
Absolute monocyte count (×10^9^/l)	0·5 [0·3–0·8]	1065	0·8 [0·5–1·4]	847	<0·0001	1·0 [0·6–1·6]	517	0·5 [0·3–0·9]	154	0·6 [0·3–0·9]	174	<0·0001
Lactate (mM)	2·2 [1·6–3·0]	1052	5·6 [3·1–8·3]	851	n/a	4·8 [2·9–8·8]	521	6·4 [5·7–8·0]	154	4·3 [2·7–8·1]	171	0·0045
Patients with lactic acidosis (>5 mmol/l) (%)	0 (0%)	1052	482 (56%)	851	n/a	248 (48%)	521	154 (100%)	154	80 (47%)	171	<0·0001
Oximetry saturation (%)	99 [97–100]	1052	97 [94–99]	849	n/a	97 [95–99]	519	98 [96–99]	154	96 [94–98]	171	0·069
Patients with hypoxia (SpO_2_ <90%) (%)	0 (0%)	1052	43 (5%)	849	n/a	22 (4%)	519	6 (4%)	154	10 (6%)	171	0·77
Patients with respiratory distress (%)	74 (7%)	1078	518 (61%)	855	<0·0001	310 (59%)	522	78 (51%)	154	125 (72%)	174	0·51
Cerebral malaria (%)	0 (0%)	1078	174 (20%)	855	n/a	0 (0%)	522	0 (0%)	154	174 (100%)	174	n/a
Deaths (%)	0 (0%)	1078	48 (4·5%)	855	n/a	10 (1·9%)	522	5 (3·3%)	154	33 (19·0%)	174	<0·0001
Host phenotypes for receptors of parasite cytoadhesion ligands: median, [IQR] or n (%)												
Group A	266 (24·7%)	1077	252 (29·5%)	854	<0·0001	140 (26·8%)	522	50 (32·7%)	153	61 (35·1%)	174	0·016
Group AB	36 (3·3%)		65 (7·6%)			49 (9·4%)		5 (3·3%)		10 (5·7%)		
Group B	238 (22·1%)		190 (22·2%)			122 (23·4%)		33 (21·6%)		34 (19·5%)		
Group O	537 (49·9%)		347 (40·6%)			211 (40·4%)		65 (42·5%)		69 (39·7%)		
A1 positive (A1 or A1B)	226 (90·0%)	251	227 (92·3%)	246	0·47	132 (88·6%)	149	43 (100%)	43	50 (96·2%)	52	0·007
A1 negative (non-A1 and non-A1B)	25 (10·0%)		19 (7·7%)			17 (11·4%)		0 (0%)		2 (3·8%)		
Monocyte CD54 MFI	290 [189–404]	1018	201 [118–298]	805	<0·0001	174 [98–264]	495	286 [188–406]	143	225 [135–311]	162	<0·0001
Monocyte CD36 MFI	216 [121–341]	1031	134 [69–229]	819	<0·0001	135 [72–222]	503	149 [72–271]	147	112 [58–231]	164	0·97
Platelet CD36 MFI	140 [82–215]	1046	141 [80–208]	828	0·79	129 [77–192]	507	166 [100–243]	149	146 [86–230]	167	0·0009

UM, uncomplicated malaria; SM, severe malaria; IQR, interquartile range; MCV, mean corpuscular volume; RBC, red blood cell; iRBC, infected red blood cell; MFI, median fluorescence intensity; n/a Not applicable for comparison.

* Severe Malaria Anaemia (SMA) with or without lactic acidosis but excluding cerebral malaria (CM).

† Isolated Lactic Acidosis (LA) excludes SMA or CM.

‡ Cerebral Malaria (CM) includes all cases of CM with or without LA or SMA.

§ non-SMA refers to isolated LA combined with CM, as defined above.

**Fig. 5:**
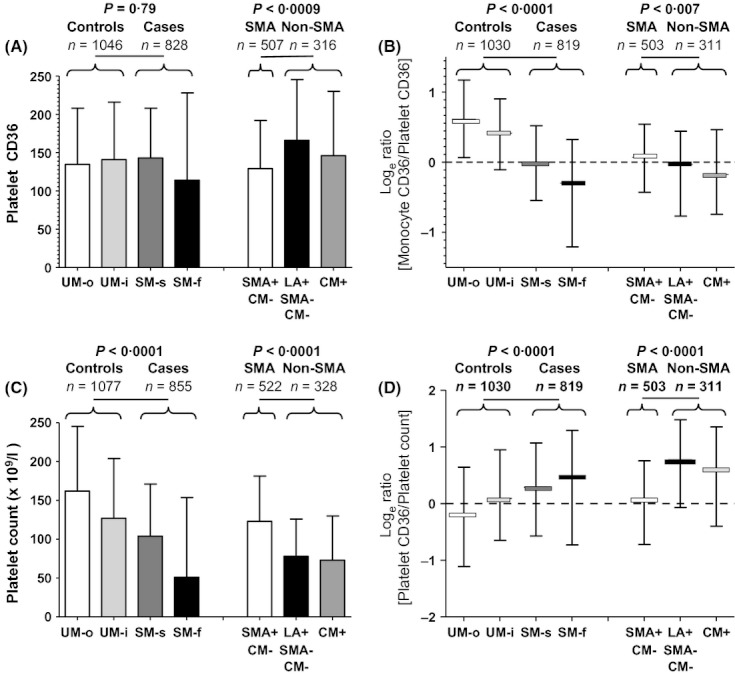
CD36 Expression and platelet count in patients with malaria. (A) The median and inter-quartile range of platelet CD36 expression (measured as median fluorescence intensity through flow cytometry) is shown. (B) A progressive decline in the median and inter-quartile range of the log_e_ of the ratio of monocyte-to-platelet CD36 expression according to disease severity is shown. (C) Increasing disease severity and cases of CM or LA are associated with progressive thrombocytopenia. (D) A progressive rise in the median and inter-quartile range of the log_e_ of the ratio of platelet CD36 expression to platelet count with disease severity and with CM and LA is shown. UM-o, uncomplicated malaria treated as an outpatient; UM-i, uncomplicated malaria treated as an inpatient; SM-s, severe malaria, survivor; SM-f, severe malaria, fatal; SMA, severe malaria anaemia; LA, lactic acidosis; CM, cerebral malaria.

SM cases reflected severe malaria anaemia (SMA, *n* = 558), lactic acidosis (LA, *n* = 482), and cerebral malaria (CM, *n* = 174) with some expected overlap (Fig 1B). Hypoxia was present in 43 children and was the only SM manifestation in five. For the analysis of SM syndromes in Table I and Fig 5, SM cases were classified into three non-overlapping groups as follows: all CM (*n* = 174); SMA with or without LA (SMA, *n* = 522); and isolated LA without either SMA or CM (isolated LA, *n* = 154), Table I, Fig 1B. Because we hypothesize that SMA is a syndrome characterized by less receptor-dependent microvascular occlusion than the syndromes of CM and isolated LA, these severe syndromes were compared accordingly. SMA patients were significantly younger, had evidence of greater splenomegaly, and a lower burden of parasitaemia (Table I). In contrast, children with CM or isolated LA had lower platelet, leucocyte and monocyte counts, and higher blood lactate levels. The case fatality rate (CFR) was nearly ten-fold higher for those with CM compared with SMA (19% vs. 2%, *P* < 0·0001).

### ABO blood group

The distribution of ABO types varied substantially between UM vs. SM, χ^2^ = 29·57, *P* < 0·0001. Group A or AB prevalence was 9·1% higher in SM (37·1%) compared with UM (28·0%), *P* < 0·0001; while group O prevalence was 9·3% higher in UM (49·9%) compared with SM (40·6%), *P* < 0·0001. This was further reflected in a consistent gradient of changing ABO proportions across severity subgroups (UM-o, UM-i, SM-s, SM-f) (Fig 2A). Group A or AB was more likely than group O to have a fatal versus uncomplicated outcome [OR = 2·27, 95% confidence interval (CI) 1·21–4·28, *P* = 0·047]. In logistic regression analysis, group A or AB remained significantly associated with SM (OR = 1·50, 95% CI 1·2–2·1, *P* = 0·0008) after controlling for the effect of age, gender, HbS, platelet count, leucocyte count, hyperparasitaemia, monocyte CD54, monocyte CD36 and platelet CD36 (Fig 3). The ABO distribution was also vastly different between enrolled children with SM (Table I) and the 180 000 healthy adults who donated blood in 2008 to the nationalized Uganda Blood Transfusion Service (A: 24·9%, B: 20·9%, AB: 4·5%, O: 49·7%; personal communication, Dr Dorothy Kyeyune, Nakasero Blood Transfusion Service) (Fig 2B).

The distribution of ABO groups remained significantly different in SM for SMA versus non-SMA cases (χ^2^ = 10·36, *P* < 0·0157) (Fig 2A). Although the proportion of group O patients did not vary within the three SM subgroups, (40%, 42%, 40%), the non-O distributions consisted of more group A patients among those with CM or isolated LA versus SMA (33·9% vs. 26·8%, *P* = 0·033), and correspondingly more group B (B or AB) among those with SMA [32·8% (23·4% + 9·4%) vs. 25·1% (20·5% + 4·6%), *P* = 0·021]. When group A or AB cases were further sub-classified according to the presence or absence of the A1 antigen, a larger proportion of the patients with CM or isolated LA were represented among those positive for A1 (*P* = 0·016, Fig 2C). The A1 antigen was present in 97·9% of non-SMA subjects, while A1-negative variants were present in a 5-fold higher proportion (11·4%) in SMA (OR 5·99, 95% CI 1·35–26·56) (Table II in supporting information).

### Monocyte CD54 (ICAM1)

Monocyte levels were significantly higher in SMA compared with the other severe malaria syndromes, *P* < 0·0001 (Fig 4A). Median monocyte CD54 (ICAM1) expression per cell was significantly lower in SMA compared with other SM syndromes (*P* < 0·0001) or in cases compared with controls (*P* < 0·0001) (Fig 4B).

### Monocyte CD36 and platelet CD36

Monocyte CD36 levels were significantly higher in UM versus SM (*P* < 0·0001) (Fig 4C). Among cases, however, median monocyte CD36 values did not differ by syndromes. Platelet CD36 expression, in contrast, did not differ between SM and UM, but was significantly higher among those with CM or isolated LA compared with SMA (*P* = 0·0009), (Fig 5A). Given higher relative monocyte CD36 in UM and higher relative platelet CD36 in non-SMA, the monocyte-to-platelet CD36 ratio best accounted for the differential tissue expression effects of CD36 (Fig 5B). This ratio decreased in proportion with increasing disease severity (*P* < 0·0001), and with CM and isolated LA.

Thrombocytopenia is recognized by the WHO as a manifestation of severe malaria (WHO, 2000). The present study confirmed this association (Table I) and further found that platelet counts were significantly lower in syndromes associated with cytoadhesion (CM and isolated LA) compared with SMA (Fig 5C). We found that the relationship between platelet CD36 expression and platelet count, reflecting CD36 expression per platelet, varied with both severity and syndrome type. UM-o patients had the highest platelet counts with lowest observed platelet CD36 expression; whereas CM and isolated LA had lowest platelet counts with highest platelet CD36 expression (Fig 5D).

### Host receptor phenotypes in fatal and uncomplicated malaria

Table II compares host receptor phenotypes between children with fatal or uncomplicated malaria outcomes. The prevalence of group A or AB was significantly higher (47·9% vs. 28·0%, *P* = 0·005) in SM-f versus UM, while monocyte ICAM1 and CD36 expression were significantly higher in UM compared with SM-f (*P* < 0·0001).

**Table II:** Host receptor phenotypes in fatal and uncomplicated malaria

	Prevalence in (or value in) fatal malaria	Prevalence in (or value in) uncomplicated malaria	*P*-value
Group A or AB	23/48 47·9%	302/1077 28·0%	0·005
Monocyte CD54: median MFI	196 (95–281) *N* = 44	290 (189–404) *N* = 1018	<0·0001
Monocyte CD36: median MFI	69 (32–160) *N* = 44	215 (121–340) *N* = 1031	<0·0001
Platelet CD36: median MFI	114 (50–222) *N* = 47	140 (82–215) *N* = 1046	0·148
Log e (monoCD36/pltCD36): median	−0·29 (−1·20 to +0·30) *N* = 44	+0·48 (−0·07 to +0·95) *N* = 1030	<0·0001

MFI, median fluorescence intensity; monoCD36, monocyte CD36; pltCD36, platelet CD36.

## Discussion

This prospective study of *P. falciparum* infection demonstrated a relationship between the expression of host receptors for ligands of cytoadhesion and disease severity. The malarial syndromes marked most by hypoperfusion, namely CM and LA, are of particular significance owing to their unrivalled case fatality rates (Marsh *et al*, 1995). Cytoadhesion results in sequestration and rosetting of iRBCs (Ho *et al*, 1991; Pongponratn *et al*, 1991; Silamut *et al*, 1999; Doumbo *et al*, 2009), impaired microvascular perfusion, and endothelial damage (van der Heyde *et al*, 2006). Despite parasiticides, cytoadhesion continues until iRBC structures are cleared (Hughes *et al*, 2010). We studied children because of their higher relative risk of fatal outcomes, their comparatively less-developed adaptive immunity, and their least-selected distribution of severity-mitigating traits on initial presentation. Our hypothesis was focused not on the absence or presence of malaria (in the matter of its invasion), but on host factors associated with clinical severity.

Factors associated with mortality prior to reproductive age are those most likely to shape the human genome. While previous studies (Table III in supporting information) (Rowe *et al*, 2007; Fry *et al*, 2008) have suggested an association between ABO and severity, we document for the first time an increased odds ratio of death associated with A or AB types, providing clinical evidence to substantiate malaria as an evolutionary driver of human ABO blood group distributions (Cserti & Dzik, 2007). We found the relative risk of fatal malaria to UM was 2·3-fold higher for group A or AB versus group O. Among SM cases, the ratio of A to B types was 1·5-fold higher in the syndromes of CM and isolated LA when compared with SMA. We also identified for the first time that group A1 (the wild-type with the highest A antigen expression (Berneman *et al*, 1991)) was over-represented in the severe malaria syndromes of CM and isolated LA. We propose that genetic selection against the primordial A1 antigen results in enrichment for either group O in association with UM, or for weaker non-A1 types or group B in association with syndromes such as SMA.

This study is novel for the simultaneous analysis of the major malaria cytoadhesion receptors found on erythrocytes, leucocytes, and platelets together with established clinical factors across a spectrum of disease severity. In a logistic regression model, the odds ratio for the harm of blood group A or AB or the benefit of group O remained significant even after controlling for numerous clinical factors including HbS (Fig 3). Consistent with prior reports on its protective effect (Aidoo *et al*, 2002; Williams *et al*, 2005; Jallow *et al*, 2009; Bougouma *et al*, 2012; Gong *et al*, 2012), we observed significantly lower levels of parasitaemia in those with HbS. However, perhaps due to the low overall prevalence of HbS in our study population, the proportion of HbS-positive patients was not significantly different in UM vs. SM. In addition, our study design focused on malaria severity, and excluded uninfected controls or asymptomatic parasitaemia. Prior reports have highlighted the role of HbS during these earlier stages of infection (Gong *et al*, 2012), which our study was not designed to assess. For example, Crompton *et al* (2008) concluded that the presence of HbS was associated with delayed onset of disease, and Verra *et al* (2007) proposed that the protective effect of HbS was related to enhanced innate immunity, while Gong *et al* (2012) inferred both innate and acquired immunity played age-dependent protective roles. Other studies have discovered that the protective effects of HbS may also result from mechanisms including, but not limited to, cytoadhesion (Cholera *et al*, 2008; Ferreira *et al*, 2011).

That malaria is likely to be changing global distributions of ABO types (Cserti & Dzik, 2007; Cserti-Gazdewich *et al*, 2011) is supported not only by our severity or survival associations, but also by the large differences noted between our SM cases and healthy Ugandan adult blood donors. Because the burden of malaria surged over the last several decades despite control efforts, nearly all Ugandan blood donors have successfully overcome prior and frequent exposures to *P. falciparum*, especially in rural areas with higher endemicity than present in our study. Thus, malaria selection may be a plausible explanation for the shift in ABO distributions that we observed within a single nation and generation.

ICAM1 is expressed on the endothelium and may serve as a sequestration receptor for iRBC. The level of expression of ICAM1 on monocytes has had an uncertain overall role in malaria. Monocyte ICAM1 may indirectly enhance iRBC immune clearance but may conversely co-localize with iRBC in CM (Stevenson & Riley, 2004; Baratin *et al*, 2007). We found a surprising and significant association between lower monocyte ICAM1 expression in SMA compared with other severe syndromes. This low expression may reflect down-regulation (or inhibition of upregulation) (Skorokhod *et al*, 2004) of ICAM1 by haemozoin (Schwarzer *et al*, 1998), which has been linked to the pathogenesis of SMA (Perkins *et al*, 2011). Alternatively, enhanced clearance of monocytes with high ICAM1 expression may have occurred. Congruent with data from Kenya (Novelli *et al*, 2010), we found that the clinical factors associated with SMA were younger age, more palpable splenomegaly, and higher monocyte counts.

The effects of CD36 in malaria have likewise been particularly controversial. On monocytes, CD36 supports phagocytosis of iRBC (McGilvray *et al*, 2000); while on integumentary tissues (such as the fat or skin), it detains iRBC and may enhance vector re-transmission. Cytoadhesion in CM has been proposed to result from bridging iRBCs to endothelial cells via CD36 expression on platelets (Pain *et al*, 2001; Bridges *et al*, 2010). For example, Wassmer *et al* (2004) demonstrated *in-vitro* that binding of iRBCs to brain endothelial cells was mediated by platelets and was CD36-dependent. Because genetic studies (Fry *et al*, 2009; Cserti-Gazdewich *et al*, 2011) have provided conflicting conclusions, we measured CD36 expression phenotypically rather than inferring expression from genotypes. We demonstrated that monocyte CD36 expression was significantly higher in UM versus SM, and that platelet CD36 expression was significantly higher in severe cytoadhesive syndromes. We suggest that the most clinically informative measure of CD36 is the ratio of its monocyte (beneficial) to platelet (harmful) expression, with ratio values inversely proportional to severity and with low ratios associated with CM and isolated LA. This is in keeping with our previously proposed model of the clinical significance of CD36 resulting from differential tissue expression (Cserti-Gazdewich *et al*, 2011). Genetic alterations assumed to diminish CD36 expression across tissues may in fact have inhomogeneous effects, as shown by zygosity studies where heterozygous deficiency resulted in substantially decreased expression on platelets compared with monocytes (Imai *et al*, 2002), thus potentially maximizing the benefit of a “carrier” state for CD36 deficiency (Cserti-Gazdewich *et al*, 2011). As with HbS in malaria, CD36 polymorphisms may exhibit a heterozygous advantage.

Thrombocytopenia is a hallmark of *Plasmodial* infections, with several mechanisms unique in *P*. *falciparum*, including platelet binding to iRBCs and endothelial cells (de Mast, 2011; de Mast *et al*, 2010). We confirm an association between thrombocytopenia and SM, as well as CM and isolated LA, syndromes speculated to result in part from cytoadhesion.

The strengths of our study include its mechanism-driven design of case evaluation, large-volume accrual, completeness of case reporting, laboratory blinding, and use of validated assays for host receptors of adhesion ligands. In this largest prospective study of malaria outcomes according to host adhesion receptors, limitations nevertheless included: not characterizing parasite-encoded adhesins or performing adhesion assays with patient-derived iRBC isolates (Ochola *et al*, 2011); inability to directly measure endothelial ligand expression; and not having quantified ICAM1 and CD36 expression through illness and recovery. We do not yet know which factors control expression of ICAM1 and CD36, nor how well their expression on blood cells correlates with endothelial cells. We therefore cannot conclude whether the observed relative expression of host receptors of adhesion were the causes or associated effects of outcomes. Despite attempts to match UM and SM for age prospectively, while not assessing for pre-existing humoral malaria immunity, we saw the expected enrichment of older (and possibly adaptively protected) children among those with UM. Nevertheless, our results remained significant after adjusting for age. Finally, the study's single site location, despite serving as a national referral centre, may have emphasized adaptations that are nevertheless regional and not necessarily generalizable to other malaria-endemic areas.

In summary, we have demonstrated that host phenotypes for receptors of parasite cytoadhesion ligands associate with the clinical severity of malaria, as well as with high mortality manifestations. Specifically, SMA exhibited low ICAM1 expression, whereas CM or isolated LA demonstrated a high prevalence of group A1 and an elevated ratio of platelet-to-monocyte CD36. Informed by the molecular interactions of parasite and human, the measurable “panel” of ABO type, monocyte ICAM1 and CD36 expression, and platelet CD36 expression translates clinically. The determination of ABO type, an unchanging attribute and a categorical measure of the host, is of particular appeal because of its ease of testing in resource-limited settings, and its significance at the level of malaria mortality. The observation that ABO is an independent risk factor for survival among children with malaria provides the most compelling explanation to date for evolution away from the A1 wild-type and towards the weaker subgroups of A, group B or AB, and most importantly group O. Finally, these results invite approaches to malaria treatment that transcend the challenge of developing new cost-prohibitive drug therapies to which the parasite may also gain resistance. Re-directing our focus on co-evolutionary interactions between host and parasite suggests new lines of research. For example, the use of “universal” group O RBCs rather than type-specific RBCs may prove especially advantageous for group A1 patients. Because *in vitro* studies document that soluble blood group substances can block the formation of iRBC rosettes (Barragan *et al*, 2000), competitive inhibition of cytoadhesion using plasma-sourced soluble A/B substances or other targeted inhibitors may merit clinical investigation. Medicine may thus strive to recapitulate the advantages already gained by natural selection.
