# Supplementary material for: Cytoadherence in paediatric malaria: ABO blood group, CD36, and ICAM1 expression and severe Plasmodium falciparum infection
Source: Br J Haematol. 2012 Aug 22;159(2):223–36. doi: 10.1111/bjh.12014 (PMC3470923; doi:10.1111/bjh.12014)
Supplement: Supplementary file 1 [file bjh0159-0223-SD1.pdf]

Cytoadherence in Paediatric Malaria: ABO Blood Group, CD36, and ICAM-1 Expression and Severe *Plasmodium falciparum* Infection

Supplementary Materials

Table of Contents

Study Websites 2

Methods
Study Setting *2*
Enrolment Details *3*
Characterization of P falciparum Parasitaemia 3
Consent Form *4*
Data Collection 5
HIV Testing *5*
Cytoadhesion Receptor Phenotyping
ABO 6
Flow Cytometry 6
Logistic Regression Analysis 7

Results
ABO 7
Flow cytometry 12

Figures
Figure 1: Study Accrual History 3
Figure 2: Correlation of Screening Score for Parasitaemia with Reference Laboratory Calculated Quantitative Parasitaemia 4
Figure 3: Stability of Measured Expression of Monocyte CD54 (ICAM-1), Monocyte CD36, and Platelet CD36 (Glycoprotein IV) According to Sample Age 6
Figure 4a: Raw Distribution of Median Fluorescence Intensity for Monocyte CD54 (ICAM-1), Monocyte CD36, and Platelet CD36 (Platelet Glycoprotein IV) 12
Figure 4b: Log Transformation of Median Fluorescence Intensity for Monocyte CD54 (ICAM-1), Monocyte CD36, and Platelet CD36 (Platelet Glycoprotein IV) 12

Tables
Table I: Flow of Enrollees Analyzed for Logistic Regression 7
Table IIa ABO Data by Severity 8
Table IIb ABO Data by Clinical Syndromes 9
Table IIc ABO Case Fatality Rates 10
Table IId ABO and Survival 10
Table IIe Haematologic Features by O vs A Types in Severe Malaria 10
Table III Summary of Literature on ABO Effect in Malaria 11

Additional Acknowledgements 13

References 14

Study Websites:
www.cd36malaria.org
http://clinicaltrials.gov/ct2/show/NCT00707200

Methods:

Study Setting:
P falciparum remains highly endemic in 95% of Uganda, a mainland sub-Saharan east-central African nation. Despite control measures, the malaria burden has not fallen to the same extent that it has in other areas with lower baseline transmission intensities, island geographies, or more developed public health infrastructures.(Yeka*, et al* 2011) Kampala, the most populous urban district and capital city of Uganda, is equatorial (00°18'49"N, 32°34'52") at a low-to-moderate altitude (1,190m) with a highly heterogeneous (low to medium-high) malaria endemicity.(Clark*, et al* 2010, Keiser*, et al* 2004) Peak incidences occur following the two rainy seasons (March to May, and September to November), with the highest prevalences in proximity with standing water.(Clark*, et al* 2010, Odongo-Aginya*, et al* 2005, Staedke*, et al* 2003) Exposed residents receive 5-50 infective bites per year, and *P falciparum* accounts for 90-98% of all malaria infections.(Zaramba 2005-2010) In children admitted to Mulago Hospital, malaria accounts for 30-50% of outpatient visits, 35% of hospital admissions, and ~10% of hospital deaths.(Achan*, et al* 2011, Idro and Aloyo 2004, Yeka*, et al* 2011) Screening work on children from Kampala has found a prevalence of microscopic *P falciparum* of 17% in asymptomatic children, and 34% by polymerase chain reaction (PCR). It is only the former method (with a roughly 10-fold lower sensitivity(Moody 2002)) which reflects a higher burden of parasitaemia and a 5-fold higher rate of subsequently symptomatic malaria, with 82% experiencing clinical sequelae within 20 weeks.(Nsobya*, et al* 2004) As such, this particular study setting (with respect to both endemicity and subject age) is enriched for symptomatic malaria and does not emulate the high rates of asymptomatic parasitaemia seen in other parts of sub-Saharan Africa, where >90% of adults may have molecularly detectable parasitaemia.(Bottius*, et al* 1996)

In Kampala, Mulago Hospital is a 1,500+ bed government-owned and operated national referral centre, as well as the nation's largest teaching hospital, affiliated with and located on the campus of Makerere University College of Health Sciences (MUCHS). The Department of Paediatrics and Child Health admits 20,000 children annually through the Acute Care Unit [ACU] from the 100 emergency patients assessed daily. Complementing the four rotating general paediatric wards which receive patients admitted from the ACU are the Resuscitation Unit, a neonatal Special Care Unit, and a Nutrition Unit for the severely malnourished. In addition to the inpatient care areas, the department operates several general and specialized outpatient clinics throughout the week. There are 34 staff paediatricians with other healthcare workers, including post-graduate medical officers, nurses, and laboratorians. Most of the clinical services provided by the department are free of charge to the patients in accordance with government policies on cost sharing in public hospitals.

Enrolment Details:

Figure 1: Study Accrual History

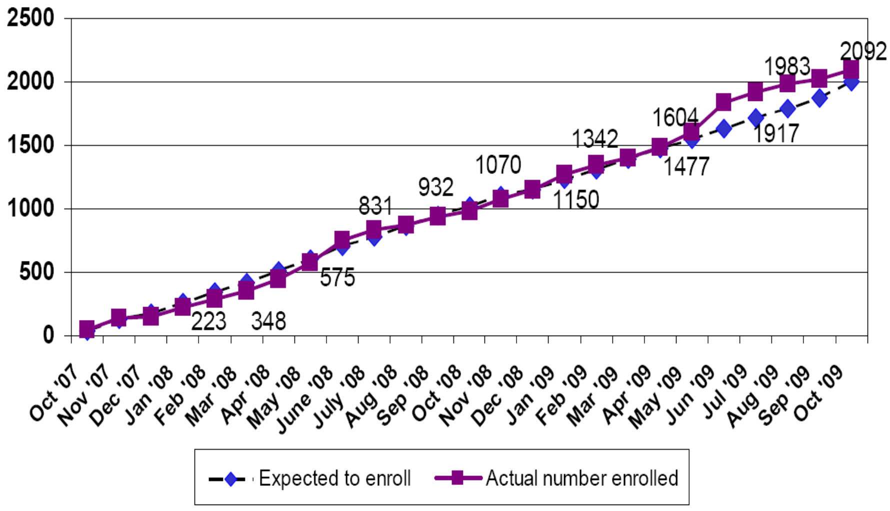

Each enrolled patient was assigned a consecutive unique patient number (UPN). To prevent transcription errors, a tear-off sheet of grouped UPN labels was pre-printed for each consecutive subject. These labels were applied to paper forms, blood tubes, laboratory reports, peripheral blood films and archived samples.

Patients who presented to hospital at any time were eligible, with effort made to balance the number of cases with controls. Two full-time research officers (AD, CM) evaluated those ACU patients with *P falciparum* malaria identified on the fingerprick screening blood smears, with assessments for eligibility, enrolment, consent, and active case characterization occurring through weekday-daytime shifts (08:00 – 17:00) and after-hours shifts (weeknights 17:00 – 24:00 and Saturdays 08:00 – 17:00).

The accrual plan was to enroll 2000 patients over the 2 year study period, for approximately 3 enrolments per day. Throughout this period, enrolments were maintained on the projected schedule (Fig 1).

Characterization of P. falciparum Parasitaemia

Consideration of patients for enrollment required a positive screening test for malaria using a stained thick film finger-stick specimen reviewed by a trained laboratory ACU technician. Screening films were scored on a four point semi-quantitative scale of parasitaemia. Enrolled patients then had both a thick and thin film prepared, air dried, and promptly transferred to an independent reference parasitology laboratory in Kampala (Molecular Research Laboratory [MoLab], Makerere University – University of San Francisco). The slides were stained and examined by two independent parasitologists as per standard technique.(World Health Organization. 2010) Both were blinded to the clinical attributes or case classification of subjects. Thin films were used for speciation, and thick films were used for parasitaemia quantitation. The number of parasitized red cells observed on thick films was indexed to the number of observed leukocytes, counting at least 200 leukocytes per film. The actual leukocyte count rather than an assumed leukocyte count of 8 $x10^9$/L was used to calculate the level of parasitaemia.(Jeremiah and Uko 2007) The results were averaged for the two readers.

We noted a significant difference in calculated parasitaemia when assumed versus actual leukocyte counts were used (paired t-test, $p < 2x10^{-8}$), attributable to higher median leukocyte counts of 11 $x10^9$/L in severe malaria (SM) compared with 8 $x10^9$/L in uncomplicated malaria (UM). A high level of agreement was noted for both speciation and quantitative parasitaemia between the two reading parasitologists, with the mean variation in parasite densities being

1.6% ($p = 0.30$). There was a correlation between the final quantitative parasitaemia as determined at the MoLab and the semi-quantitative screening score examination in the ACU ($r^2 = 0.1989$, $p < 0.0001$) (Fig 2).

Figure 2: Correlation of Screening Score for Parasitaemia with Reference Laboratory Calculated Quantitative Parasitaemia

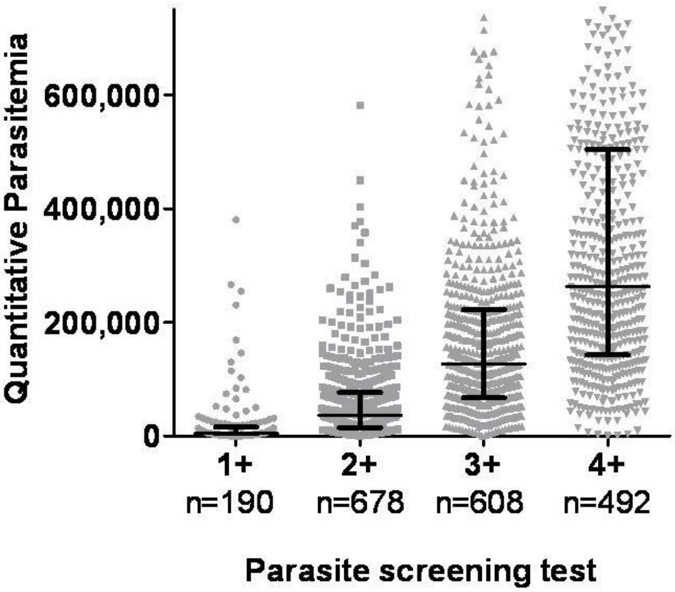

Consent Form

The consent form was developed with assisted review from research ethicists in Toronto, Canada and Kampala, Uganda, in accordance with Good Clinical Practice Guidelines. Two consent forms were implemented, with the first dedicated to authorizing real-time clinical and pre-defined laboratory review until discharge as per the CPM study protocol, and the second to the option of specimen archiving for future unspecified proteomic and genomic analyses relevant in malaria biology. The consent form was written in English so as to be understood at a simple grade school level, with translation into Luganda, the most widely spoken language of Uganda. Validity of the translation was confirmed with back-translation to English through a third party. The research officers, fluent in both Luganda and English, were trained in the administration and documentation of informed consent from the parents or legal guardians of the paediatric subjects. If the child had capacity to give assent, this was documented. Due to the prevalence of illiteracy, consent was verbally established, and either signatures (impressed through the multi-copy NCR forms) or thumbprints (upon all copies) were accepted. Parents/guardians retained a copy of consent forms with supplementary study summary pamphlets and investigator contact information. Of 2092 CRFs, 99.6% had written signatures, with 6 thumbprint-only forms. There were three cases of presumed consent, as permitted by pre-study dispensation from all certifying ethics boards in the contingency of death from malaria (in a final denominator of 48 fatalities).

Data Collection

Each enrolled patient was evaluated and treated by a physician experienced in malaria care, and all available clinical resources were used to assess for the presence of other conditions. Clinical data were first collected on a hard-copy Case Report Form (CRF). The CRF (available at www.cd36malaria.org) was divided into 8 sections: consent and sample control; dates of enrollment and discharge; demographics; history and physical; laboratory values; cerebral malaria; hypoxia and lactic acidosis; and transfusion and malaria therapy. Apart from entry of laboratory results, free text entries were not used. After each subject's discharge, data were transferred from the CRF form to a digital CRF. Regular (fortnightly x 55) conference calls reviewed logistics, tabulated accrual statistics, monitored the extent of on-pace hard-copy to digital data transfer. The accuracy of transfer of hard-copy CRF data into the digital database was audited by sampling each 25th CRF on a quarterly basis. The digital CRF (prepared in FileMaker Pro 9.0 v 1, Santa Clara, CA) mirrored the paper CRF, with features to prevent transcription errors including inability to re-use the UPN which identified each subject; inability to leave required fields unfilled; inability to record duplicate data for all single value responses; radio buttons; range limits on data fields; and required digital signatures. Paper and digital CRFs were kept in a secure, locked environment during the study. At the conclusion of the study, all paper CRFs (with patient name and UPN) were shipped to the study headquarters for secure storage, and for use to resolve any data discrepancies found in the database.

The electronic study database in Excel (Microsoft, Redmond, WA) was prepared from exports of the digital CRF. The database underwent extensive testing for data integrity, consistency, and accuracy. Scrutiny included range value testing, missing data testing, logical tests on data consistency across fields, and multiple comparisons with the original paper CRF record. Strict version control was used. All data analyses were done on a single version of a finalized corrections dataset.

HIV Testing

Enrolled patients (n= 2092) were subject to HIV testing at Mulago Hospital as per policies of healthcare worker (rather than client) – initiated HIV counseling and testing (HCT) within the Mulago – Mbarara Teaching Hospitals' Joint AIDS Program (MJAP),(Wanyenze*, et al* 2008) with assistance from the existing Makerere University – Johns Hopkins University (MU-JHU) Research Collaboration. The local protocol used a commercially-available enzyme-linked screening assay (Clearview® STAT-PAK®, Inverness Medical, Louisville CO, USA). Negative screening tests were re-tested with a second assay (Uni-Gold™ Recombigen® HIV, Trinity Biotech PLC, Wicklow, Ireland), and positive screening tests confirmed with a second enzyme-linked assay (Murex HIV Ag/Ab Combination, Abbott Diagnostics Division, Dartford, UK; or Vironistika® HIV Uni-Form II Ag/Ab, bioMérieux, Lyon, France). In Kampala, 740 patients underwent testing, including 2 with a reported history of HIV. The 2 HIV-co-infected patients and 5 others were found to be HIV-positive (1.0%). Archived fresh frozen citrated plasma aliquots from the remaining 1,331 who were not tested in Kampala (either because of opt-out preferences or local test kit unavailability), and not already excluded from intended analysis (due to absence of confirmatory *P falciparum* parasitaemia [n=35] or non-conformance with pre-defined age eligibility [n=1]), were then subject to HIV testing with a high-sensitivity commercial assay for antigen and antibody at Mount Sinai Hospital's Microbiology Laboratory in Toronto, Canada (Architect System HIV Ag/Ab Combo, Abbott Diagnostics Division, Weisbaden, Germany). Another 38 subjects (2.9%) were identified as HIV-positive, for a total of 45 HIV+ subjects excluded from analysis. Of the 733 subjects identified as HIV-negative by laboratory

testing Kampala, 0.5% of samples were randomly re-assessed in Toronto to validate the negative result.

Cytoadhesion Receptor Phenotyping

ABO:

ABO blood groups were determined locally on peripheral blood collected in EDTA- anticoagulated tubes. ABO Blood Group Reagents (Anti-A BioClone®, Anti-B BioClone®, Anti-D for Slide and Modified Tube Tests, ORTHO® Anti-A1 Lectin; Ortho-Clinical Diagnostics, Inc, Raritan, NJ) were applied to whole blood for direct haemagglutination typing according to manufacturer's directions.

Flow Cytometry:

The EDTA sample was then transported from Mulago Hospital to the Joint Centre for Clinical Research (JCRC) located < 5km away for testing by flow cytometry and sickle haemoglobin screening in the T-Cell Laboratory.

Samples were stained and tested by flow cytometry (FACScan, Becton-Dickinson, Franklin Lakes, NJ) and the results analyzed using commercial software (FloJO, Tree Star, Inc, Ashland, OR) by the director of the laboratory (IS). Four-channel commercial beads (CaliBRITE beads and APC-beads, Becton-Dickinson) were used to calibrate the cytometer with each run. In addition, a second set of phycoerythrin coated beads (QuantiBRIGHT, Becton-Dickinson) were used to control for channel fluorescence in the photomultiplier specific for the readout of CD36 measurements. The evaluation of surface expression of ICAM-1 on monocytes,(Cserti-Gazdewich*, et al* 2010) and CD36 on platelets and monocytes,(Cserti-Gazdewich*, et al* 2009, Dzik*, et al* 2010) had been based on our previously validated and described methods.

In previous work and in this study, we validated the stability of flow cytometric assay readings over sample storage time, in days (Fig 3).

Figure 3: Stability of Measured Expression of Monocyte CD54 (ICAM-1), Monocyte CD36, and Platelet CD36 (Glycoprotein IV) According to Sample Age

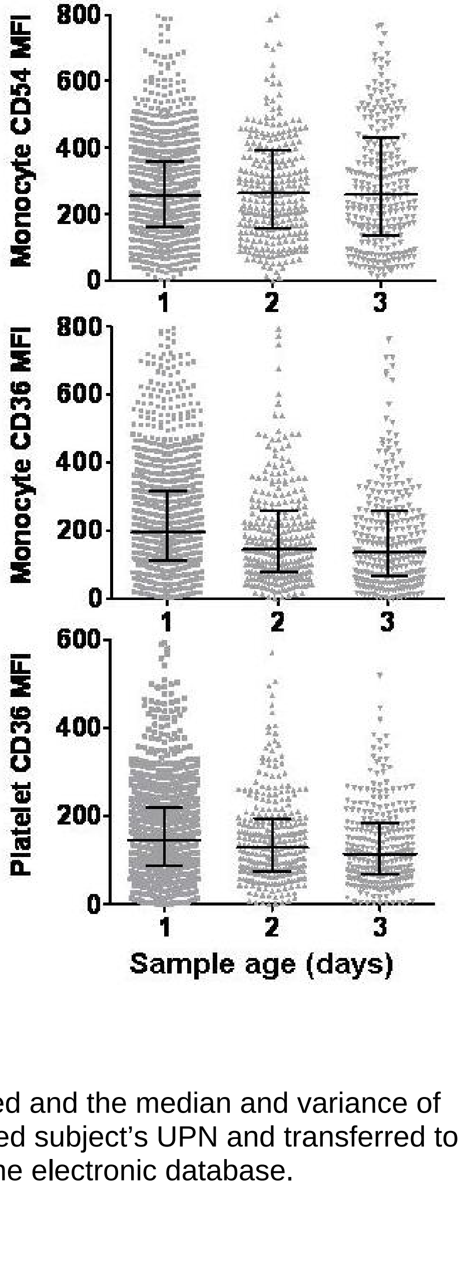

Results of flow cytometry, including numbers of cells assayed and the median and variance of fluorescence intensity, were indexed according to the enrolled subject's UPN and transferred to the study headquarters where they were incorporated into the electronic database.

Logistic Regression Analysis

Logistic regression analysis (Stata ver 11.1, Stata Corp, College Station, TX) was used to analyze factors associated with malaria severity as follows: From the 1933 SM and UM patients, full data for every logistic variable of interest was present for 1777, with one or more missing variable fields for 156 patients (8.1%) (Table I). Most instances of incomplete data were due to absence of data on flow cytometry (n=111 patients lacking one or more readings, where 110 lacked monocyte CD54, 83 lacked monocyte CD36, and 59 lacked platelet CD36), as a result of an interruption of a shipment of reagents from North America. The reagent interruption was confirmed to not have systematically excluded unique subsets of cases or controls. A total of 39 subjects had missing data on quantitative parasitaemia levels but had strongly positive results for parasitaemia screening. Nine patients had incomplete data for blood counts or blood groups. Sickle cell testing results were available for 1871 subjects.

Table I: Flow of Enrollees Analyzed for Logistic Regression

Factor	Number with result for factor
Age	1933
Gender	1933
Flow cytometry	1823
Haemoglobin S	1871
Hyperparasitaemia	1894
Leukocyte count	1925
ABO group	1931
Platelet count	1932
Complete data for all factors	1777

Results:

ABO

Results for ABO grouping according to severity of disease and according to severe malaria syndrome are listed in Table IIa and Table IIb respectively. Case fatality rates according to ABO are show in Table IIc and the ABO proportions among survivors are shown in Table IId. Haematologic features among group O and group A cases with severe malaria are shown in Table IIe.

Table IIa: ABO Data by Severity

ABO	all enrolled	all analyzed	Primary comparison		Severity Spectrum				
			SM (cases)	UM (controls)	Deaths	SM-s	Non-SM, Non-UM	UM-i	UM-o
A	541/2009 26.9%	518/1931 26.8%	252/854 29.5%	266/1077 24.7%	19/48 39.6%	233/806 28.9%	23/78 29.5%	188/761 24.7%	78/316 24.7%
Non-AO/AA (AB or O or B)	1468/2009 73.1%	1413/1931 73.2%	602/854 70.5%	811/1077 75.3%	29/48 60.4%	573/806 71.1%	55/78 70.5%	573/761 75.3%	238/316 75.3%
Non-any A (O or B)	1362/2009 67.8%	1312/1931 67.9%	537/854 62.9%	775/1077 72.0%	25/48 52.1%	512/806 63.5%	50/78 64.1%	553/761 72.7%	222/316 70.3%
B	447/2009 22.2%	428/1931 22.2%	190/854 22.2%	238/1077 22.1%	7/48 14.6%	183/806 22.7%	19/78 24.4%	174/761 22.9%	64/316 20.3%
Non-BO/BB (AB or O or A)	1562/2009 77.8%	1503/1931 77.8%	664/854 77.8%	839/1077 77.9%	41/48 85.4%	623/806 77.3%	59/78 75.6%	587/761 77.1%	252/316 79.7%
Non-any B (O or A)	1456/2009 72.5%	1402/1931 72.6%	599/854 70.1%	803/1077 74.6%	37/48 77.1%	562/806 69.7%	54/78 69.2%	567/761 74.5%	236/316 74.7%
A or AB	647/2009 32.2%	619/1931 32.1%	317/854 37.1%	302/1077 28.0%	23/48 47.9%	294/806 36.5%	28/78 35.9%	208/761 27.3%	94/316 29.7%
B or AB	553/2009 27.5%	529/1931 27.4%	255/854 29.9%	274/1077 25.4%	11/48 22.9%	244/806 30.3%	24/78 30.8%	194/761 25.5%	80/316 25.3%
AB	106/2009 5.3%	101/1931 5.2%	65/854 7.6%	36/1077 3.3%	4/48 8.3%	61/806 7.6%	5/78 6.4%	20/761 2.6%	16/316 5.1%
O	915/2009 45.5%	884/1931 45.8%	347/854 40.6%	537/1077 49.9%	18/48 37.5%	329/806 40.8%	31/78 39.7%	379/761 49.8%	158/316 50.0%
Non-O	1094/2009 54.5%	1047/1931 54.2%	507/854 59.4%	540/1077 50.1%	30/48 62.5%	477/806 59.2%	47/78 60.3%	382/761 50.2%	158/316 50.0%
A1 positives (A1 or A1B)	471/517 91.1%	453/497 91.1%	227/246 92.3%	226/251 90.0%	16/16 100.0%	211/230 91.7%	18/20 90.0%	155/173 89.6%	71/78 91.0%
A1-negatives (A2 or A2B)	46/517 8.9%	44/497 8.9%	19/246 7.7%	25/251 10.0%	0/16 0%	19/230 8.3%	2/20 10.0%	18/173 10.4%	7/78 9.0%
Non-A1 (A2 or A2B or O or B)	1408/1879 74.9%	1356/1809 75.0%	556/783 71.0%	800/1026 78.0%	25/41 61.0%	531/742 71.6%	52/70 74.3%	571/726 78.7%	229/300 76.3%

Table IIb: ABO Data by Clinical Syndromes

ABO	all CM	all LA	all SMA	CM (no SMA, no LA)	LA (no SMA, no CM)	SMA (no CM, no LA)	SMA (no CM)	all CM plus LA without SMA
A	61/174<br>35.1%	148/481<br>30.8%	154/558<br>27.6%	25/82<br>30.5%	50/153<br>32.7%	71/274<br>25.9%	140/522<br>26.8%	111/327<br>33.9%
Non-AO/AA<br>(AB or O or B)	113/174<br>64.9%	333/481<br>69.2%	404/558<br>72.4%	57/82<br>69.5%	103/153<br>67.3%	203/274<br>74.1%	382/522<br>73.2%	216/327<br>66.1%
Non-any A<br>(O or B)	103/174<br>59.2%	298/481<br>62.0%	352/558<br>63.1%	52/82<br>63.4%	98/153<br>64.1%	180/274<br>65.7%	333/522<br>63.8%	201/327<br>61.5%
B	34/174<br>19.5%	96/481<br>20.0%	131/558<br>23.5%	17/82<br>20.7%	33/153<br>21.6%	75/274<br>27.4%	122/522<br>23.4%	67/327<br>20.5%
Non-BO/BB<br>(AB or O or A)	140/174<br>80.5%	385/481<br>80.0%	427/558<br>76.5%	65/82<br>79.3%	120/153<br>78.4%	199/274<br>72.6%	400/522<br>76.6%	260/327<br>79.5%
Non-any B<br>(O or A)	130/174<br>59.2%	350/481<br>72.8%	375/558<br>67.2%	60/82<br>73.2%	115/153<br>75.2%	176/274<br>64.2%	351/522<br>67.2%	245/327<br>74.9%
A or AB	71/174<br>40.8%	183/481<br>38.0%	206/558<br>36.9%	30/82<br>36.6%	55/153<br>35.9%	94/274<br>34.3%	189/522<br>36.2%	126/327<br>38.5%
B or AB	44/174<br>25.3%	131/481<br>27.2%	183/558<br>32.8%	22/82<br>26.8%	38/153<br>24.8%	98/274<br>35.8%	171/522<br>32.8%	82/327<br>25.1%
AB	10/174<br>5.7%	35/481<br>7.3%	52/558<br>9.3%	5/82<br>6.1%	5/153<br>3.3%	23/274<br>8.4%	49/522<br>9.4%	15/327<br>4.6%
O	69/174<br>39.7%	202/481<br>42.0%	221/558<br>39.6%	35/82<br>42.7%	65/153<br>42.5%	105/274<br>38.3%	211/522<br>40.4%	134/327<br>41.0%
Non-O	105/174<br>60.3%	279/481<br>58.0%	337/558<br>60.4%	47/82<br>57.3%	88/153<br>57.5%	169/274<br>61.7%	311/522<br>59.6%	193/327<br>59.0%
A1 positive<br>(A1 or A1B)	50/52<br>96.2%	135/146<br>92.5%	144/161<br>89.4%	21/22<br>95.5%	43/43<br>100.0%	62/69<br>89.9%	132/149<br>88.6%	93/95<br>97.9%
A1 negative<br>(A2 or A2B)	2/52<br>3.8%	11/146<br>7.5%	17/161<br>10.6%	1/22<br>4.5%	0/43<br>0%	7/69<br>10.1%	17/149<br>11.4%	2/95<br>2.1%
Non-A1<br>(A2 or A2B or O or B)	105/155<br>67.7%	309/444<br>69.6%	369/513<br>71.9%	53/70<br>75.7%	98/141<br>69.5%	187/249<br>75.1%	350/482<br>72.6%	203/296<br>68.6%

Table IIc: ABO Case Fatality Rates (CFR)

	A	non-A	B	non-B	A or AB	B or AB	AB	O	non-O	O or B	A1	non-A1
CFR	19/541<br>3.5%	29/1468<br>2.0%	7/447<br>1.6%	19/541<br>2.6%	23/647<br>3.6%	11/553<br>2.0%	4/106<br>3.8%	18/915<br>2.0%	30/1094<br>2.7%	25/1362<br>1.8%	16/471<br>3.4%	0/46<br>0%
95% CI	2.0 – 5.1%	1.3 – 2.7%	0.4 – 2.7%	1.8 – 3.4 %	2.1 – 5.0 %	0.8 – 3.2 %	0.1 – 7.4%	1.1 – 2.9%	1.8 – 3.7%	1.1 – 2.6%	1.8 – 5.0%	n/a

Table IId: ABO and Survival

	All analyzed subjects (INPUT)	Deaths	All analyzed survivors (OUTPUT)	All SM subjects (INPUT)	All surviving SM subjects (OUTPUT)
**A**	518/1933 (26.8 %)	19/48 (39.6 %)	499/1885 (26.5 %)	252/806 (28.9 %)	233/806 (28.9 %)
**AB**	101/1933 (5.2 %)	4/48 (8.3 %)	97/1885 (5.1 %)	65/806 (7.6 %)	61/806 (7.6 %)
**B**	428/1933 (22.2 %)	7/48 (14.6 %)	421/1885 (22.3 %)	190/806 (22.7 %)	183/806 (22.7 %)
**O**	884/1933 (45.8 %)	18/48 (37.5 %)	866/1885 (45.9 %)	347/806 (40.8 %)	329/806 (40.8 %)
**A1**	453/497 (91.1 %)	16/16 (100 %)	227/246 (92.3 %)	227/246 (92.3 %)	211/230 (91.7 %)
**A2**	44/497 (8.9 %)	0/16 (0 %)	19/246 (7.7 %)	19/246 (7.7 %)	19/230 (8.3 %)

Table IIe: Haematologic Features by O vs A Types in Severe Malaria

	O (n=347)	A or AB (n=317)	p-value
Hb (g/L)	52 ± 24	49 ± 20	0.045
Platelets (x 10^3/µL)	124 ± 102	135 ± 111	0.16
Quantitative parasitaemia (/µL)	236,000 ± 331,000	191,000 ± 283,000	0.076
Lactate (mM)	6.3 ± 3.7	6.3 ± 3.7	1.0

Supplemental Literature Summary:

A summary of prior studies examining the association of ABO blood groups with severe malaria is shown in Table III.

Table III. Summary of Literature on ABO Effect in Malaria.

Author (Year)	Study site	Study size			Definition of cases	Severe Malaria (n) / ABO Category (N)					
						A (A/AB) vs non-A (O/B)			O vs non-O (A/B/AB)		
		Total	Controls (UM)	Cases (SM)		A (A or AB) n / N	Non-A (O or B) n/N (%)	OR (95% CI)	O n/N (%)	Non-O (A/B/AB) n/N (%)	OR (95% CI)
Al-Yaman et al (1995)	Papua New Guinea	253	156	97	CM	43/118 (36.4%)	54/135 (40.0%)	0.860 (0.518-1.428)	44/97 (45.4%)	53/156 (34.0%)	1.61 (0.96-2.71)
Fischer and Boone (1998)	Zimbabwe	271	251	20	Coma	9/65 (13.9%)	11/206 (5.3%)	2.85 (1.15-7.06)	8/139 (5.8%)	12/132 (9.1%)	0.611 (0.25-1.51)
Lell et al (1999)	Gabon	200	100	100	HP, SMA, other WHO SM	30/45 (66.7%)	70/155 (45.2%)	2.43 (1.22-4.83)	54/118 (45.8%)	46/82 (56.1%)	0.66 (0.38-1.16)
Pathirana et al (2005)	Sri Lanka	243	163	80	CM, SMA, MSOF	39/87 (44.8%)	41/156 (26.3%)	2.28 (1.31-3.95)	19/97 (19.6%)	61/146 (41.8%)	0.35 (0.19-0.63)
Rowe et al (2007)	Kenya & Mali	472	309	163	CM, SMA, other WHO SM	70/153 (45.8%)	93/319 (29.2%)	2.05 (1.38-3.05)	37/177 (20.9%)	126/295 (42.7%)	0.35 (0.23-0.54)
Fry et al (2008)	Gambia, Malawi, Kenya	3995	1903	2092	SMA, CM, RD	670/1164 (57.6%)	1422/2831 (50.2%)	1.34 (1.17-1.54)	896/1828 (49.0%)	1196/2167 (55.2%)	0.78 (0.70-0.88)
Tekeste et al (2010)	Ethiopia	210	140	70	CM, SMA, shock	39/92 (42.4%)	31/118 (26.3%)	2.07 (1.16-3.68)	16/130 (12.3%)	54/80 (67.5%)	0.35 (0.19-0.67)
This report (2012)	Uganda	1931	1077	854	CM, SMA, LA, HO	317/619 (51.2%)	537/1312 (40.9%)	1.52 (1.25-1.84)	347/884 (39.3%)	507/1047 (48.4%)	0.69 (0.57-0.83)
Totals		7575	4099	3476		1217/2343 (51.9%)	2259/5232 (43.2%)	1.42 (1.29-1.57)	1421/3420 (41.6%)	2100/4155 (50.5%)	0.73 (0.63-0.80)

Flow Cytometry

Flow cytometry results were available for 1779 patients (59 missing platelet CD36 data, 83 missing monocyte CD36 data, and 110 missing monocyte CD54 data). Distributions of the measured values for the median fluorescence intensity of monocyte CD54 (ICAM-1), monocyte CD36, and platelet CD36 are shown in Fig 4a. The same data, log-e transformed, are shown in Fig 4b.

Figure 4a: Raw Distribution of Median Fluorescence Intensity (MFI) for Monocyte CD54 (ICAM-1), Monocyte CD36, and Platelet CD36 (Platelet Glycoprotein IV)

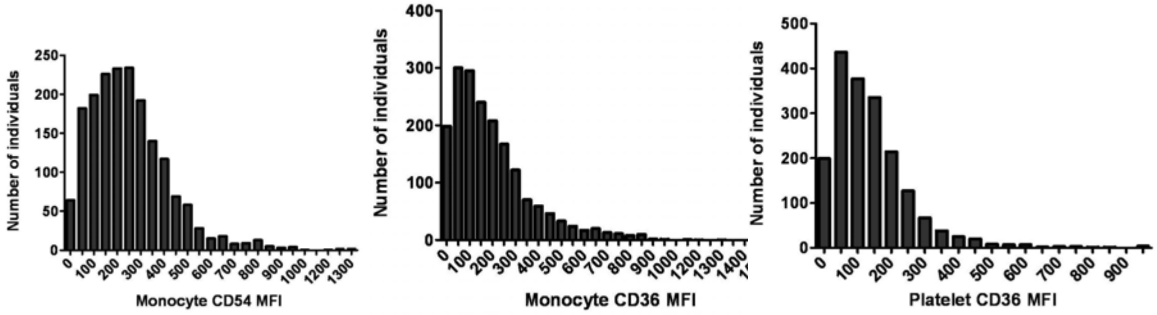

Figure 4b: Log Transformation of Median Fluorescence Intensity for Monocyte CD54 (ICAM-1), Monocyte CD36, and Platelet CD36 (Platelet Glycoprotein IV)

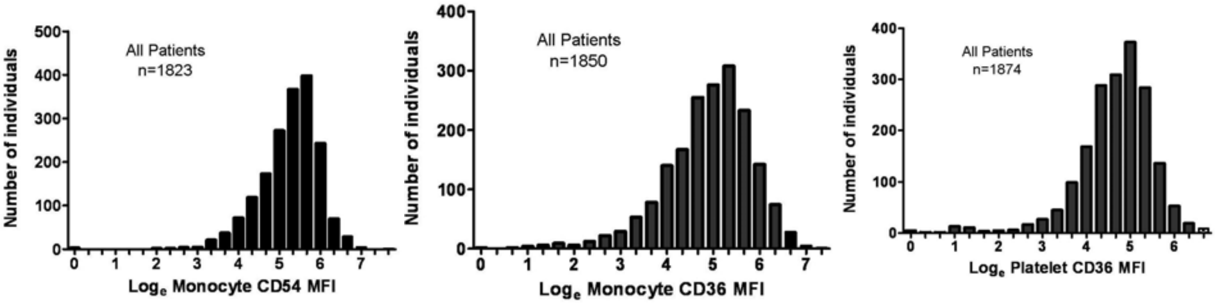

Additional Acknowledgements:

We also gratefully acknowledge the support of the following: Dr Sammuel Nsobya and the parasitology technologists of the MU-UCSF MoLab; Dr Francis Ssali and the flow cytometry technologists (Denis Akakimpa, David Baliruno, Godfrey Pimundu, Rose Nabatanzi Kitandwe, Steven Mutalya) at the Joint Clinical Research Centre Flow Cytometry Lab; Dr Sarah Kiguli-Walube (Department Head of Paediatrics at Makerere University College of Health Sciences), Dr Robert Opoka (Medical Director of Acute Care Unit, Mulago Hospital), Jolly Rubambarama (head RN at Acute Care Unit of Mulago Hospital), the malaria blood film screening staff at the Acute Care Unit (Edson Sabuni, Josephine Birungi, Rehema Namwanje, Timothy Pande, David Balamusani, Stephen Ikodi, Moses Kizito, and Vincent Sekibala), specimen transport chain management in Kampala (Abdu Mwanje); HIV testing laboratory staff (Dr Tony Mazzulli and Lilian Law at Mount Sinai Hospital in Toronto); our advisors in research ethics and materials transfer (Dr Ronald Heselgrave and Leanne Ramirez at the Toronto Academic Health Sciences Network Research Ethics Board; Drs Charles Ibingira, Elly Katabira, Charles Opio, Israel Kalyesubula, and Paul Waako at Mulago Hospital; Dr Livingstone Luboobi at Makerere University); and our advisors in proposal design and implementation (Dr Mark Crowther and Nancy Heddle at McMaster University, Dr Connie Westhoff at the New York Blood Center, and Dr Kevin Kain at the McLaughlin-Rotman Centre for Global Health at the University of Toronto).

References

Achan, J., Tibenderana, J., Kyabayinze, D., Mawejje, H., Mugizi, R., Mpeka, B., Talisuna, A. & D'Alessandro, U. (2011) Case management of severe malaria--a forgotten practice: experiences from health facilities in Uganda. *PLoS ONE,* **6,** e17053.

al-Yaman, F., Genton, B., Mokela, D., Raiko, A., Kati, S., Rogerson, S., Reeder, J. & Alpers, M. (1995) Human cerebral malaria: lack of significant association between erythrocyte rosetting and disease severity. *Transactions of the Royal Society of Tropical Medicine and Hygiene,* **89,** 55-58.

Bottius, E., Guanzirolli, A., Trape, J.F., Rogier, C., Konate, L. & Druilhe, P. (1996) Malaria: even more chronic in nature than previously thought; evidence for subpatent parasitaemia detectable by the polymerase chain reaction. *Transactions of the Royal Society of Tropical Medicine and Hygiene,* **90,** 15-19.

Clark, T.D., Njama-Meya, D., Nzarubara, B., Maiteki-Sebuguzi, C., Greenhouse, B., Staedke, S.G., Kamya, M.R., Dorsey, G. & Rosenthal, P.J. (2010) Incidence of Malaria and Efficacy of Combination Antimalarial Therapies over 4 Years in an Urban Cohort of Ugandan Children. *PLoS ONE,* **5,** e11759.

Cserti-Gazdewich, C.M., Dzik, W.H., Dorn, M.E., Quagliaroli, R.O., Xu, S., Ssewanyana, I., Nayyar, R. & Preffer, F.I. (2009) Quantitation of CD36 (platelet glycoprotein IV) expression on platelets and monocytes by flow cytometry: application to the study of Plasmodium falciparum malaria. *Cytometry B Clinical Cytometry,* **76,** 127-134.

Cserti-Gazdewich, C.M., Dzik, W.H., Erdman, L., Ssewanyana, I., Dhabangi, A., Musoke, C. & Kain, K.C. (2010) Combined measurement of soluble and cellular ICAM-1 among children with Plasmodium falciparum malaria in Uganda. *Malaria Journal,* **9,** 233.

Dzik, W.H., Cserti-Gazdewich, C.M., Ssewanyana, I., Delelys, M. & Preffer, F.I. (2010) When monocytes and platelets compete: The effect of platelet count on the flow cytometric measurement of monocyte CD36. *Cytometry B Clinical Cytometry,* **78,** 81-87.

Fischer, P.R. & Boone, P. (1998) Short report: severe malaria associated with blood group. *American Journal of Tropical Medicine and Hygiene,* **58,** 122-123.

Fry, A.E., Griffiths, M.J., Auburn, S., Diakite, M., Forton, J.T., Green, A., Richardson, A., Wilson, J., Jallow, M., Sisay-Joof, F., Pinder, M., Peshu, N., Williams, T.N., Marsh, K., Molyneux, M.E., Taylor, T.E., Rockett, K.A. & Kwiatkowski, D.P. (2008) Common variation in the ABO glycosyltransferase is associated with susceptibility to severe Plasmodium falciparum malaria. *Human Molecular Genetics,* **17,** 567-576.

Idro, R. & Aloyo, J. (2004) Manifestations, quality of emergency care and outcome of severe malaria in Mulago Hospital, Uganda. *African Health Sciences,* **4,** 50-57.

Jeremiah, Z.A. & Uko, E.K. (2007) Comparative analysis of malaria parasite density using actual and assumed white blood cell counts. *Annals of Tropical Paediatrics,* **27,** 75-79.

Keiser, J., Utzinger, J., Caldas de Castro, M., Smith, T.A., Tanner, M. & Singer, B.H. (2004) Urbanization in sub-saharan Africa and implication for malaria control. *American Journal of Tropical Medicine and Hygiene,* **71,** 118-127.

Lell, B., May, J., Schmidt-Ott, R.J., Lehman, L.G., Luckner, D., Greve, B., Matousek, P., Schmid, D., Herbich, K., Mockenhaupt, F.P., Meyer, C.G., Bienzle, U. &

Kremsner, P.G. (1999) The role of red blood cell polymorphisms in resistance and susceptibility to malaria. *Clinical Infectious Diseases,* **28,** 794-799.
Moody, A. (2002) Rapid diagnostic tests for malaria parasites. *Clinical Microbiology Reviews,* **15,** 66-78.
Nsobya, S.L., Parikh, S., Kironde, F., Lubega, G., Kamya, M.R., Rosenthal, P.J. & Dorsey, G. (2004) Molecular evaluation of the natural history of asymptomatic parasitemia in Ugandan children. *Journal of Infectious Diseases,* **189,** 2220-2226.
Odongo-Aginya, E., Ssegwanyi, G., Kategere, P. & Vuzi, P.C. (2005) Relationship between malaria infection intensity and rainfall pattern in Entebbe peninsula, Uganda. *African Health Sciences,* **5,** 238-245.
Pathirana, S.L., Alles, H.K., Bandara, S., Phone-Kyaw, M., Perera, M.K., Wickremasinghe, A.R., Mendis, K.N. & Handunnetti, S.M. (2005) ABO-blood-group types and protection against severe, Plasmodium falciparum malaria. *Annals of Tropical Medicine and Parasitology,* **99,** 119-124.
Rowe, J.A., Handel, I.G., Thera, M.A., Deans, A.M., Lyke, K.E., Kone, A., Diallo, D.A., Raza, A., Kai, O., Marsh, K., Plowe, C.V., Doumbo, O.K. & Moulds, J.M. (2007) Blood group O protects against severe Plasmodium falciparum malaria through the mechanism of reduced rosetting. *Proceedings of the National Academy of Sciences U S A,* **104,** 17471-17476.
Staedke, S.G., Nottingham, E.W., Cox, J., Kamya, M.R., Rosenthal, P.J. & Dorsey, G. (2003) Short report: proximity to mosquito breeding sites as a risk factor for clinical malaria episodes in an urban cohort of Ugandan children. *American Journal of Tropical Medicine and Hygiene,* **69,** 244-246.
Tekeste, Z. & Petros, B. (2010) The ABO blood group and Plasmodium falciparum malaria in Awash, Metehara and Ziway areas, Ethiopia. *Malararia Journal,* **9,** 280.
Wanyenze, R.K., Nawavvu, C., Namale, A.S., Mayanja, B., Bunnell, R., Abang, B., Amanyire, G., Sewankambo, N.K. & Kamya, M.R. (2008) Acceptability of routine HIV counselling and testing, and HIV seroprevalence in Ugandan hospitals. *Bulletin of the World Health Organization,* **86,** 302-309.
World Health Organization. (2010) *Basic malaria microscopy.* WHO, Geneva.
Yeka, A., Gasasira, A., Mpimbaza, A., Achan, J., Nankabirwa, J., Nsobya, S., Staedke, S.G., Donnelly, M.J., Wabwire-Mangen, F., Talisuna, A., Dorsey, G., Kamya, M.R. & Rosenthal, P.J. (2011) Malaria in Uganda: Challenges to control on the long road to elimination I. Epidemiology and current control efforts. *Acta Tropica*.
Zaramba, S. (2005-2010) Uganda Malaria Control Strategic Plan. (ed. by Program, M.C.) Available at: http://www.rollbackmalaria.org/countryaction/nsp/uganda.pdf

.
