# Supplementary figures and images for: Cytoadherence in paediatric malaria: ABO blood group, CD36, and ICAM1 expression and severe Plasmodium falciparum infection
Source: Br J Haematol. 2012 Aug 22;159(2):223–36. doi: 10.1111/bjh.12014 (PMC3470923; doi:10.1111/bjh.12014)

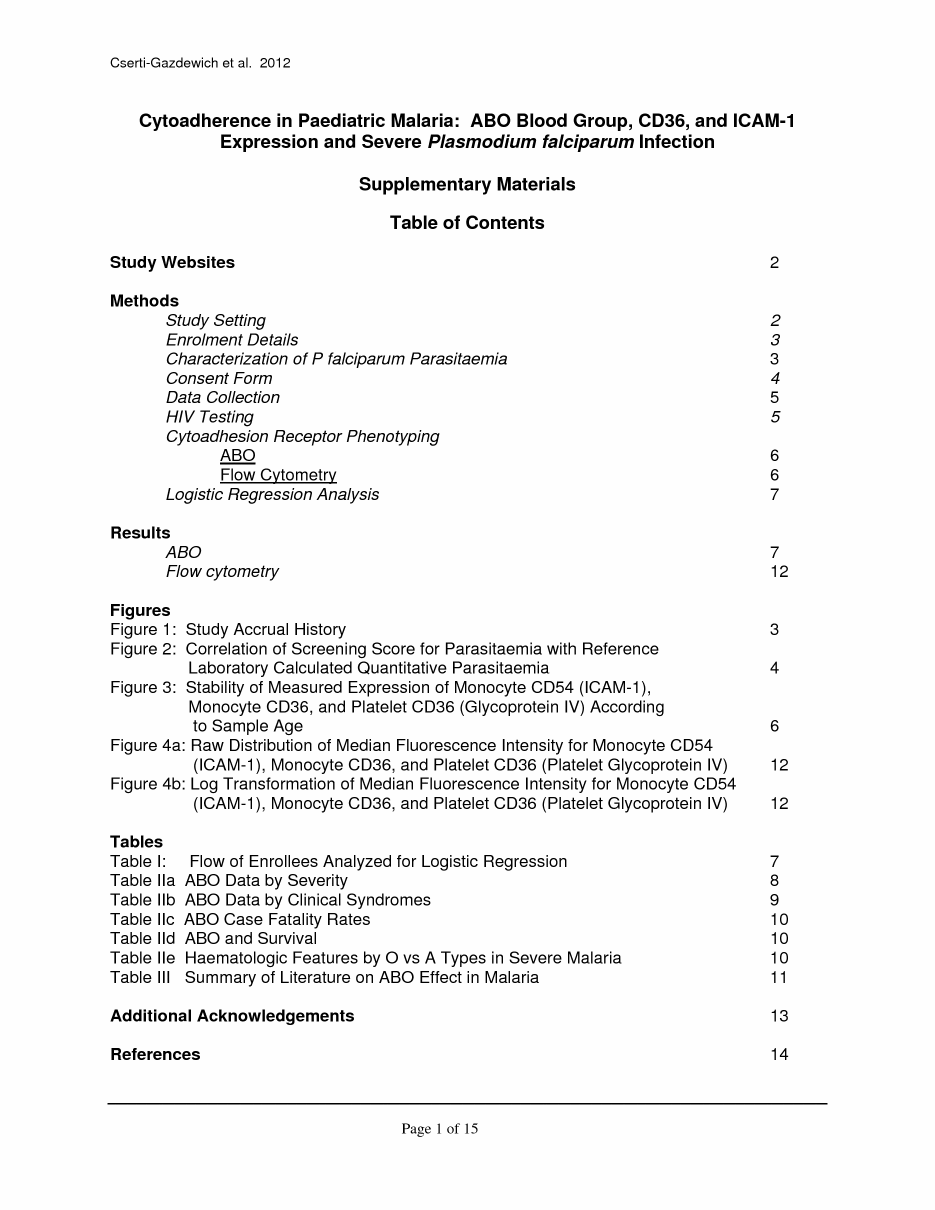

Supplement: Supplementary file 2 [file bjh0159-0223-SD2.png]
